# Agreement between Electrocardiogram and Heart Rate Meter Is Low for the Measurement of Heart Rate Variability during Exercise in Young Endurance Horses

**DOI:** 10.3389/fvets.2017.00170

**Published:** 2017-10-17

**Authors:** Augustin Lenoir, Dagmar S. Trachsel, Mohamed Younes, Eric Barrey, Céline Robert

**Affiliations:** ^1^Ecole Nationale Vétérinaire d’Alfort, Maisons-Alfort, France; ^2^CIRALE, Ecole Nationale Vétérinaire d’Alfort, Maisons-Alfort, France; ^3^CIAMS, Université Paris-Sud, Université Paris-Saclay, Orsay, France; ^4^GABI UMR1313, INRA, AgroParisTech, Université Paris-Saclay, Jouy-en-Josas, France

**Keywords:** cardiology, equine, comparison, electrocardiogram, heart rate meter, standardized exercise test

## Abstract

Analysis of the heart rate variability (HRV) gains more and more importance in the assessment of training practice and welfare in equine industry. It relies on mathematical analyses of reliably and accurately measured variations in successive inter-beat intervals, measured as RR intervals. Nowadays, the RR intervals can be obtained through two different techniques: a heart rate meter (HRM) or an electrocardiogram (ECG). The agreement and reliability of these devices has not been fully assessed, especially for recordings during exercise. The purpose of this study was to assess the agreement of two commercially available devices using the two mentioned techniques (HRM vs ECG) for HRV analysis during a standardized exercise test. Simultaneous recordings obtained during light exercise and during canter with both devices were available for 36 horses. Data were compared using a Bland–Altman analysis and the Lin’s coefficient. The agreement between the assessed HRV measures from the data obtained from the ECG and HRM was acceptable only for the mean RR interval and the mean heart rate. For the other studied measures (SDNN, root mean square of successive differences, SD1, SD2, low frequency, high frequency), the agreement between the devices was too poor for them to be considered as interchangeable in these recording conditions. The agreement tended also to be worse when speed of the exercise increased. Therefore, it is necessary to be careful when interpreting and comparing results of HRV analysis during exercise, as the results will depend upon recording devices. Furthermore, corrections and data processing included in the software of the devices affect largely the output used in the subsequent HRV analysis; this must be considered in the choice of the device.

## Introduction

Clinical evaluation of cardiac function gains more and more importance in the assessment of training status and welfare in equine industry and relies nowadays mainly on portable devices. The most commonly used devices either report the full electric activity through an electrocardiogram (ECG) or transform the registered signal in an output reporting the main electric activity (electric activity of the ventricle). This is then reported as inter-beat intervals, measured as RR intervals or even transformed to heart rate (HR) values. This later technique is known as heart rate meter (HRM). Both types of devices allow to evaluate the electric function of the heart at rest and during exercise. Nevertheless, the ECG is more complete than the HRM as it records the whole depolarization cycle while the HRM only records the time between the two main depolarization waves (RR intervals). The RR intervals can be extracted and a heart rate variability (HRV) analysis can be performed from both devices using suitable functions of the accompanying software. Both types of devices have been used in veterinary medicine for this purpose (1–10). The HR can be followed during physical activity to evaluate fitness of sport (11) or race horses (12). Further, training progresses can be monitored by serial HR measurements over a time (13–17). HRV analysis has also been used during exercise and for monitoring training in young horses (2, 3, 5–10, 14). The activation of the sympathetic nervous system by initiation of physical activity has a profound effect of HRV and overall reduces the HRV (3, 5). The occurrence of fatigue after repeated exercise has also been related to reduced HRV (6). Further, trainings over several weeks affected some measures of HRV as well as time for recovery (2, 7, 8). In humans, HRV guided training programs are under investigation and was shown to be superior to conventional training in endurance running training (18). HRV provides information on the influence of both sympathetic and parasympathetic nervous systems (19–21); it has been used to assess the effect of different stimuli on the autonomic nervous system (4, 22–28). Agreement of HRV between recording issued from ECG and HRM has also been addressed in a few studies reporting satisfactory agreement for recordings from horses at rest, standing in the stables and in liberty on pastures (29, 30). Repeatability and reliability have been assessed in few studies in horses for recording in resting condition. Overall, the repeatability was reported good, but the reliability was reported as good to poor; especially a higher day-to-day variability in short-term recording has been reported (31, 32). However, none of these studies reported agreement for recordings obtained during exercise. Therefore, the purpose of this study was to assess the agreement of the most commonly used measures of HRV obtained with the two devices.

## Materials and Methods

### Animals

The study was approved by the Animal Use and Care Committee at Alfort Veterinary School and University of Paris-Est (ComEth Anses/ENVA/UPEC; approval number 12/07/11-1). All the horse owners gave their informed consent on a written and signed document prior to any study procedures. Thirty-six young endurance horses performed a field standardized exercise test during four different measurement sessions organized between 2013 and 2014. Each horse completed the full exercise test in one session. All horses were between 4- and 6-year old. They had at least one pure-breed Arabian parent (father, mother, or both) and were registered in the Endurance breeding program of the Association Nationale Française du Cheval Arabe pur sang et demi-sang (ACA), which means they were bred and specifically trained for endurance competitions.

### Data Collection

**Figure 1:**
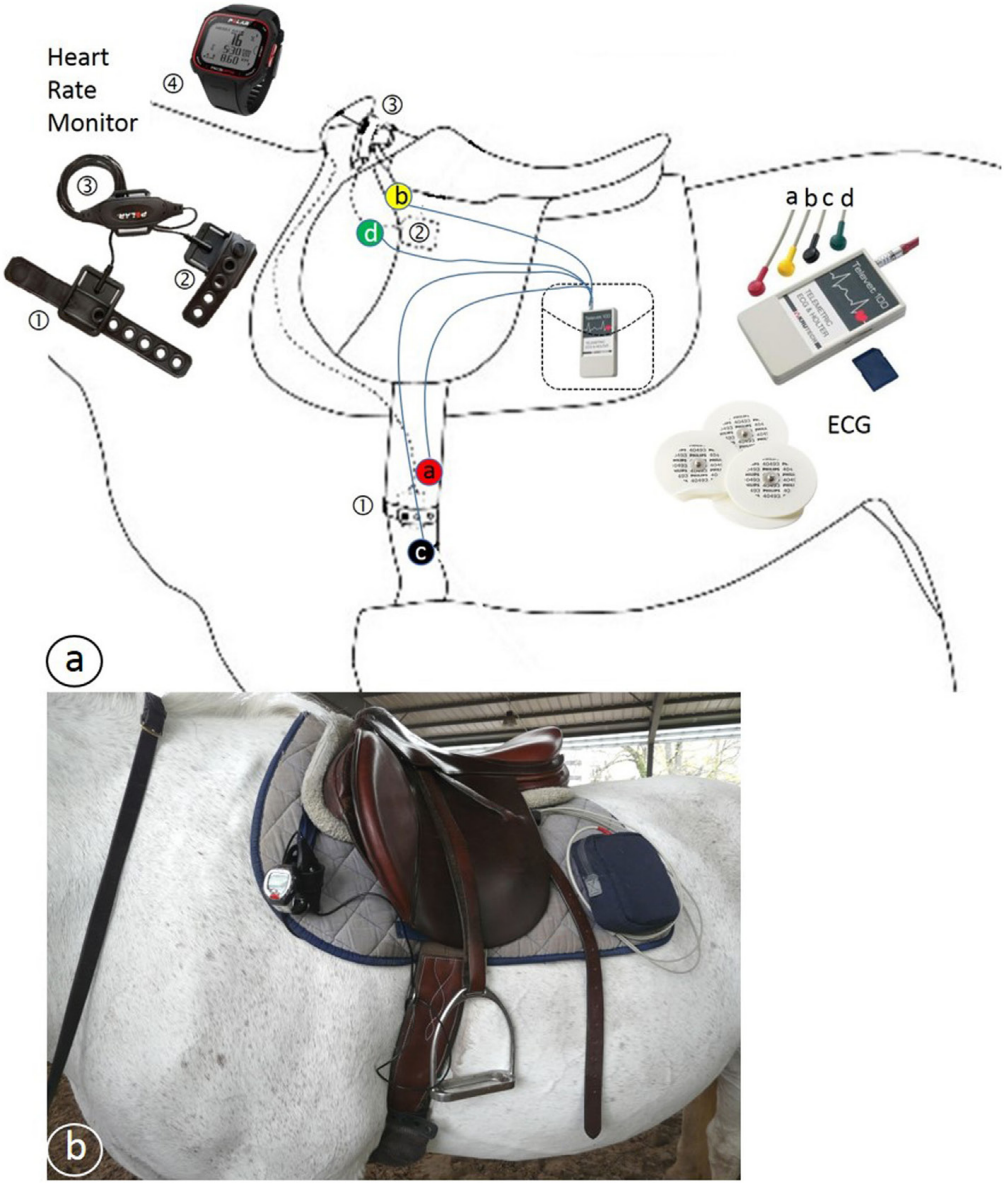
**(A)** Schematic drawing of the position of the Polar^®^ heart rate meter (HRM) and Televet^®^ electrocardiogram (ECG) equipment. ① and ② = HRM electrodes, ③ HRM transmitter, ④ HRM wrist receiver, a, b, c, and d = ECG electrodes connected by wires to the ECG receiver and transmitter placed in a pocket. **(B)** Photo of a horse with the equipment in place.

A general clinical examination was performed before exercise. The gait of the horse was assessed by trotting in hand in a straight line over 30 m to exclude any lameness. Before starting the exercise, the two different devices for heart rate monitoring were placed on the horse and the registrations were started at the same time. These were a telemetric ECG (Televet 100^®^, Jørgen Kruuse, Denmark) and an HRM (Polar S810, Polar Electro Öy, Finland). The electrodes of the ECG were placed according to the positions proposed by Zucca et al. (33) (Figure 1), and linked with a wire to a registering box in the saddle pad. For the HRM, one electrode was placed under the saddle just behind the withers on the left side and the other one was placed on the left side over the heart under the belt.

### Exercise Test

The test consisted in three standardized phases including a 15-min warm-up at walk and trot (I), followed by a 20 km/h canter phase (II) of 15 min duration for the 4-year old, 30 min for the 5-year old, 45 min for the 6-year old, and (III) a 22–25 km/h gallop phase on 500 m (34). The ECG was removed with the saddle at the end of this test between 5 and 10 min after the end of the exercise so that the riders can groom and cool their horse. A further 30 m straight line trotting in hand was performed to assess locomotion after exercise. Cardiac recovery was still monitored with the HRM for 30 min.

### Data Analysis

**Figure 2:**
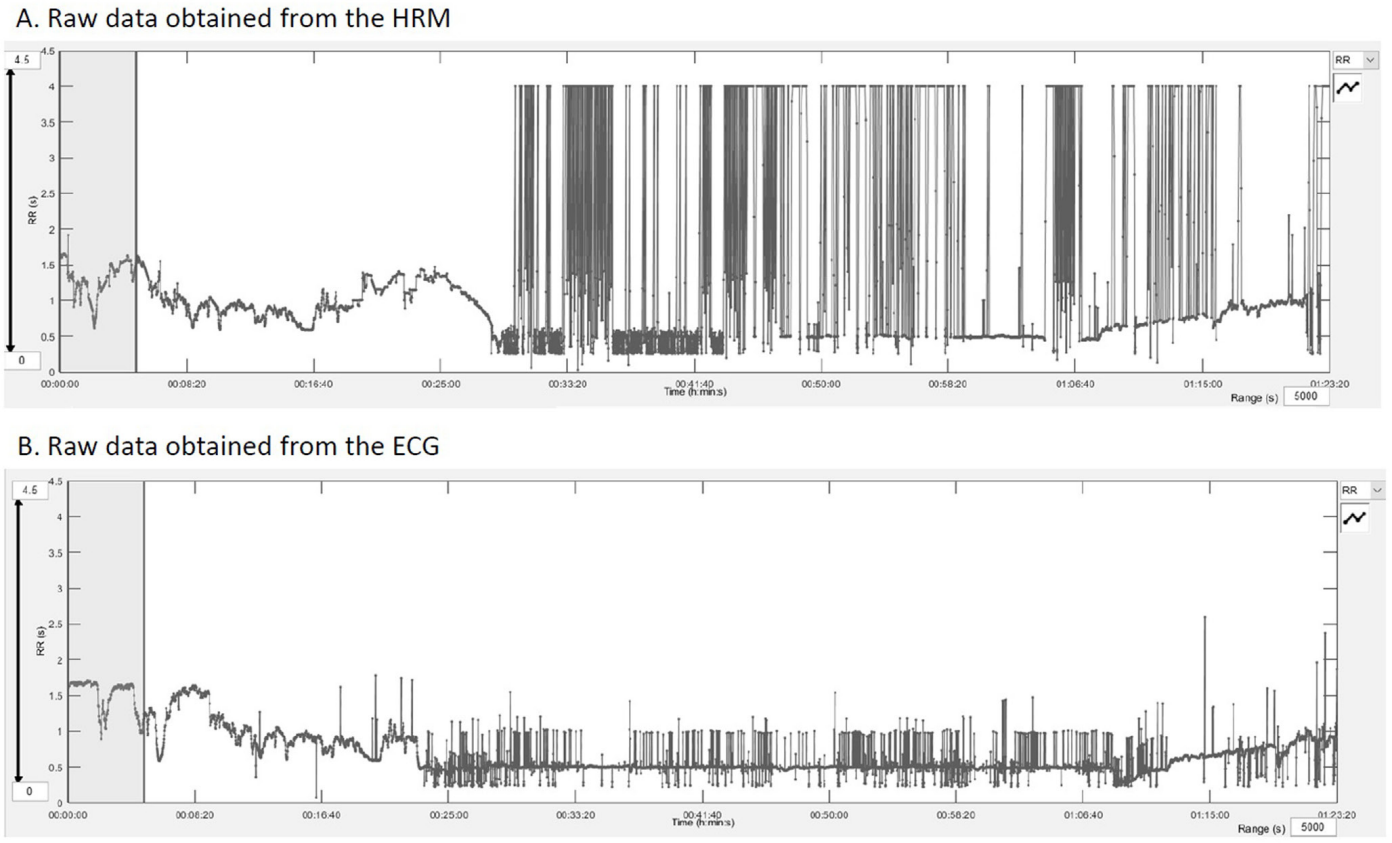
RR intervals outputs obtained after downloading the raw data into the Kubios HRV^®^ software showing the quality of the raw data and the extent of artifacts. **(A)** RR intervals in seconds registered by the HRM over the entire exercise test. **(B)** Represents the same data from the electrocardiogram recordings.

After the exercise test, the recordings were transferred to a Windows^®^-computer by Bluetooth^®^. The first step of analyze was performed offline with the reading software belonging to each device. For the HRM, the Polar Pro Trainer 5^®^ software was used to correct the data with the set up “very high” filter power and a minimal protection zone of “1 beat per minute (bpm).” This setting was chosen to have a minimum of corrections by the Polar software, in order not to correct and flatten the recording twice and, therefore, minimize the loss of variability in the HR. After visual assessment of the data, each HRM recording (with an average duration of 90 min) was then exported as RR intervals in milliseconds to a text file. If the corrected curve was too erratic and didn’t allow to see the different parts of the exercise test, the recording was removed from analysis. For the ECG, the Televet 100 5.0.0^®^ software was used, and the raw data were visually assessed. Parts of the registration with severe artifact due to loss of electrodes for example were excluded from the analyses. For the remaining ECG data, RR intervals were calculated by the software by setting the RR deviation recognition at 30% as suggested by the manufacturer. The RR intervals were subsequently exported in milliseconds as a text file. For comparison of the recordings of the two devices, further processing of the data was necessary (Figure 2). For this, the exported RR intervals from both devices were corrected by a home-made Excel^®^ algorithm. This algorithm was programmed to recognize the five types of registration errors that have been defined in veterinary medicine (21, 35). This includes errors as erratic values (I); RR intervals that are abnormally long (II); or short (III) directly followed by a short or long interval, RR-intervals corresponding to the double or triple of the precedent RR-interval (IV); or extremely short successive intervals (V). As the aim was to compare the devices, no effort was made to classify the identified errors as physiological or not. The recognition limit was set as a variation superior to 30% with the previous or following RR interval.

**Figure 3:**
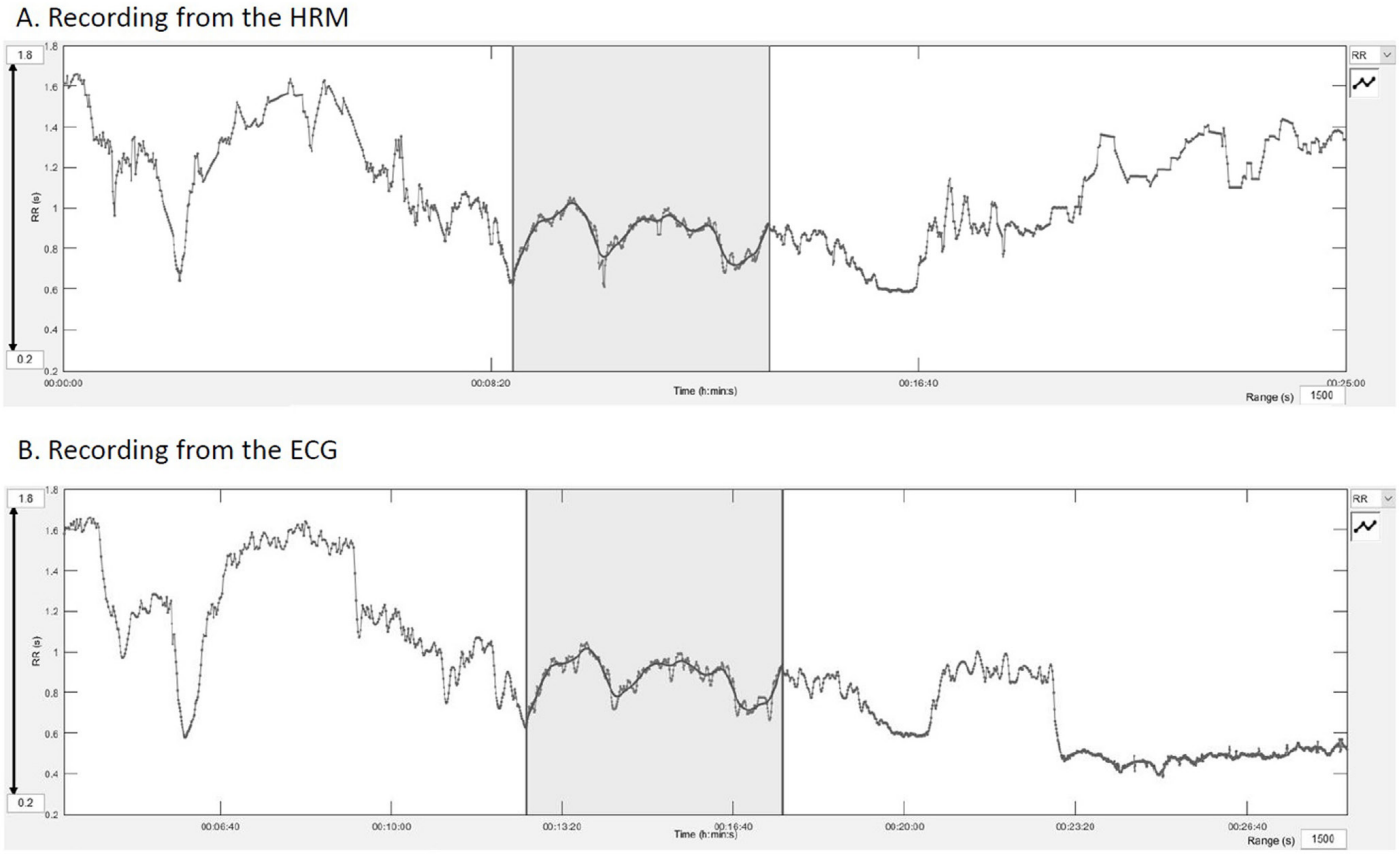
Manual synchronization from a common spike on the Kubios^®^ software. **(A)** Recording of the HRM, the gray area represents the analyzed part, **(B)** recording of the electrocardiogram.

After automatized correction of the entire datasets with the Excel^®^ algorithm, the RR text file was imported into the Kubios HRV^®^ software (University of Eastern Finland) for analysis of the HRV. Two paired ECG-HRM datasets of 5 min were chosen, one from the warm-up period and one from the canter phase. The choice was made visually on a stable HR period, starting from a common spike on both recordings (ECG and HRM) (Figure 3). Trend components were removed from every dataset using the “Smoothn priors” method with a flattening parameter (lambda) of 500 ms as recommended by Tarvainen et al. (36, 37). Low frequency (LF) power was set at 0.01–0.07 Hz, and high frequency (HF) power at 0.07–0.6 Hz according to a former publication (38).

The HRV measures used for the agreement assessment were inter-beat interval defined as RR-intervals (RR), HR, SD of RR intervals defined as normal to normal intervals (SDNN), root mean square of successive differences (RMSSD); from the Poincaré plots, the axial and perpendicular dispersions from the line of identity defined as SD1 and SD2, and finally the low and high frequency power LF and HF in square millisecond and in normalized units (abbreviated as n.u.).

### Statistical Analysis

The agreement between the different series of data were assessed using a Bland–Altman analysis (39), and with a Lin’s coefficient (CC Lin) (40). For the Bland–Altman analysis, the first measure of agreement was set *a priori* at 5% of deviation from the mean value of the studied measure of HRV (i.e., the acceptable difference between the two measurement methods was 5% of the mean value of the measure of HRV). The second measure of agreement was set at 20% of deviation from the mean value of the measure of HRV (i.e., the acceptable 95% confidence interval was 20% of the mean value of the measure of HRV). These limits were set from a clinical point of view as an acceptable error according to our experience. The accepted ranges of variations for each measure are summarized in Table 1. The ECG was used as the reference for the Bland–Altman analysis.

**Table 1:** Agreement limits for the Bland–Altman analysis.

	Warm-up		Canter	
Heart rate variability (HRV) measures	Accepted range of variation for the mean difference (5% from the mean value of the HRV measure)	Accepted range of variation for the CI95 (20% from the mean value of the HRV measures)	Accepted range of variation for the mean difference (5% from the mean value of the HRV measure)	Accepted range of variation for the CI95 (20% from the mean value of the HRV measure)
RR (ms)	−25 to +25	−100 to +100	−12.5 to +12.5	−50 to +50
SDNN (ms)	−2.5 to +2.5	−10 to +10	−0.3 to +0.3	−1.2 to +1.2
HR (bpm)	−2.5 to +2.5	−10 to +10	−7 to +7	−26 to +26
RMSSD (ms)	−2 to +2	−8 to +8	−0.5 to +0.5	−2 to +2
SD1 (ms)	−1.5 to +1.5	−6 to +6	−0.25 to +0.25	−1 to +1
SD2 (ms)	−3 to +3	−12 to +12	−0.5 to +0.5	−2 to +2
LF (ms^2^)	−50 to +50	−200 to +200	−5 to +5	−20 to +20
LF (n.u.)	−2.5 to +2.5	−10 to +10	−2.5 to +2.5	−10 to +10
HF (ms^2^)	−50 to +50	−200 to +200	−5 to +5	−20 to +20
HF (n.u.)	−2.5 to +2.5	−10 to +10	−2.5 to +2.5	−10 to +10
LF/HF	−2.5 to +2.5	−10 to +10	−2.5 to +2.5	−10 to +10

*CI95, 95% confidence interval; RR, RR-intervals; HR, heart rate; SDNN, SD of RR intervals; RMSSD, root mean square of successive differences; LF, low frequency power; HF, high frequency power*.

For the CC Lin, the limits were defined as good over 0.75, moderate between 0.6 and 0.75, poor between 0.5 and 0.6 and inacceptable under 0.5 (41).

## Results

From the 36 paired recordings obtained on all included horses, 23 pairs obtained during the warm-up period and 14 pairs obtained during canter had a suitable quality for analysis. The other recordings had to be eliminated due to impossible synchronization or because artifacts were too numerous to find a period of 5 min of recording compatible with a correct analysis of the data. The elimination of recordings was completely independent of horse identity. The results for the Bland–Altman analysis are shown in Table 2 and the results for the CC Lin in Table 3.

**Table 2:** Results of the Bland and Altman analysis for recordings obtained during the warm-up phase and canter phase.

	Mean difference (CI95)	Lower border of CI95 (CI95)	Higher border of CI95 (CI95)
**Warm-up (*N* = 23)**			

**Time domain**			
RR (ms)	**7.8 (−8.41; 24)**	**−71.59 (−99.3; −43.88)**	**87.18 (59.47; 114.89)**
SDNN (ms)	−3.93 (−7.93; 0.07)	−23.52 (−30.36; −16.68)	15.67 (8.83; 22.51)
HR (bpm)	**−0.07 (−0.92; 0.78)**	**−4.24 (−5.69; −2.78)**	**4.1 (2.64; 5.55)**
RMSSD (ms)	−2.52 (−6.89; 1.85)	−23.92 (−31.39; −16.45)	18.88 (11.41; 26.35)
**Frequency domain**			
LF (ms^2^)	−296 (−576.43; −15.56)	−1,669.85 (−2,149.4; −1,190.31)	1,077.86 (598.31; 1,557.4)
LF (n.u.)	**−1.05 (−5.11; 3.01)**	−20.94 (−27.88; −14)	18.83 (11.89; 25.77)
HF (ms^2^)	−90.71 (−341.52; 160.1)	−1,319.41 (−1,748.29; −890.53)	1,137.99 (709.11; 1,566.87)
HF (n.u.)	**1.05 (−3; 5.11)**	−18.83 (−25.77; −11.89)	20.94 (14; 27.88)
**Non-linear domain**			
SD1 (ms)	−1.78 (−4.88; 1.31)	−16.95 (−22.25; −11.66)	13.38 (8.09; 18.68)
SD2 (ms)	−5.6 (−10.62; −0.58)	−30.21 (−38.8; −21.62)	19 (10.42; 27.59)

**Canter (*N* = 14)**			

**Time domain**			
RR (ms)	0.92 (−3.02; 4.86)	−14.35 (−21.1; −7.61)	16.19 (9.45; 22.93)
SDNN (ms)	−1.29 (−3.96; 1.38)	−11.63 (−16.19; −7.06)	9.05 (4.48; 13.61)
HR (bpm)	−0.21 (−1.42; 1)	−4.9 (−6.96; −2.83)	4.48 (2.41; 6.55)
RMSSD (ms)	−3.24 (−6.74; 0.27)	−16.82 (−22.82; −10.82)	10.35 (4.35; 16.35)
**Frequency domain**			
LF (ms^2^)	127.39 (−161; 415.79)	−989.55 (−1,482.71; −496.4)	1,244.34 (751.18; 1,737.49)
LF (n.u.)	4.63 (−1.48; 10.74)	−19.05 (−29.51; −8.6)	28.31 (17.86; 38.77)
HF (ms^2^)	31.6 (−111.4; 174.6)	−522.23 (−766.76; −277.7)	585.43 (340.9; 829.96)
HF (n.u.)	−4.62 (−10.69; 1.44)	−28.12 (−38.49; −17.75)	18.87 (8.5; 29.25)
**Non-linear domain**			
SD1 (ms)	−2.29 (−4.77; 0.19)	−11.9 (−16.14; −7.66)	7.32 (3.08; 11.56)
SD2 (ms)	−0.76 (−3.79; 2.26)	−12.49 (−17.66; −7.31)	10.96 (5.79; 16.14)

*Results fitting the limit of agreement are indicated in bold*.

*CI95, 95% confidence interval; RR, RR-intervals; HR, heart rate; SDNN, SD of RR intervals; RMSSD, root mean square of successive differences; LF, low frequency power; HF, high frequency power*.

**Table 3:** Results of Lin’s coefficient (CC Lin) for recordings obtained during the warm-up and the canter phase.

	Electrocardiogram mean value (CI95)	HRM mean value (CI95)	CC Lin (CI 95)
**Warm-up (*N* = 23)**			

**Time domain**			
RR (ms)	1,163.67 (1,047.85; 1,279.48)	1,171.46 (1,050.92; 1,292.01)	**0.95 (0.93; 0.97)**
SDNN (ms)	50.34 (44.9; 55.78)	46.41 (41; 51.83)	0.71 (0.53; 0.89)
HR (bpm)	56.63 (50.21; 63.04)	56.56 (49.94; 63.17)	**0.95 (0.93; 0.97)**
RMSSD (ms)	42.29 (34.54; 50.03)	39.77 (31.22; 48.32)	0.84 (0.74; 0.94)
**Frequency domain**			
LF (ms^2^)	1,407.66 (1,021.81; 1,793.52)	1,111.67 (885.26; 1,338.07)	0.61 (0.43; 0.79)
LF (n.u.)	54.32 (48.94; 59.7)	53.27 (47.92; 58.61)	0.73 (0.55; 0.91)
HF (ms^2^)	1,245.97 (889.69; 1,602.25)	1,155.26 (804.55; 1,505.97)	0.75 (0.58; 0.92)
HF (n.u.)	45.68 (40.3; 51.06)	46.73 (41.38; 52.08)	0.73 (0.55; 0.91)
**Non-linear domain**			
SD1 (ms)	29.97 (24.47; 35.46)	28.18 (22.12; 34.24)	0.84 (0.74; 0.94)
SD2 (ms)	63.78 (57.05; 70.5)	58.18 (52.09; 64.27)	0.68 (0.48; 0.88)

**Canter (*N* = 14)**			

**Time domain**			
RR (ms)	468.96 (443.46; 494.46)	469.88 (444.09; 495.66)	**0.92 (0.89; 0.95)**
SDNN (ms)	7.24 (5.49; 8.98)	5.94 (3.8; 8.09)	0.51 (0.15; 0.87)
HR (bpm)	129.93 (124.67; 135.2)	129.73 (124.35; 135.1)	**0.92 (0.89; 0.95)**
RMSSD (ms)	7.41 (5.37; 9.45)	4.17 (2.89; 5.46)	0.02 (−0.36; 0.40)
**Frequency domain**			
LF (ms^2^)	40.42 (8.1; 72.75)	167.82 (−58.49; 394.12)	0.24 (0.18; 0.30)
LF (n.u.)	54.21 (46.73; 61.68)	58.84 (52.8; 64.87)	0.73 (0.53; 0.93)
HF (ms^2^)	44.91 (7.09; 82.73)	76.51 (−23.71; 176.73)	0.16 (−0.16; 0.48)
HF (n.u.)	45.69 (38.24; 53.14)	41.07 (35.05; 47.08)	0.73 (0.53; 0.93)
**Non-linear domain**			
SD1 (ms)	5.24 (3.8; 6.68)	2.95 (2.04; 3.86)	0.02 (−0.55; 0.59)
SD2 (ms)	8.58 (6.44; 10.73)	7.82 (4.9; 10.74)	0.63 (0.35; 0.91)

*Results fitting the limit of agreement are indicated in bold*.

*CI95, 95% confidence interval; RR, RR-intervals; HR, heart rate; SDNN, SD of RR intervals; RMSSD, root mean square of successive differences; LF, low frequency power; HF, high frequency power*.

### Warm-up

Heart rate and RR intervals showed agreement within the predefined ranges on the Bland–Altman analysis and on the CC Lin. For LF (n.u.) and HF (n.u.), the results were more ambiguous. The agreement was acceptable for only the mean difference in the Bland–Altman analysis (Table 2) (i.e., the obtained mean difference was lower than the *a priori* fixed mean acceptable difference in Table 1). For all the other measures of HRV, agreement was judged inadequate based on both the Bland–Altman analysis and the CC Lin (Table 3) (i.e., the obtained agreement parameters were higher than the *a priori* fixed ones in Table 1).

### Canter Phase

Similarly to the result in the warm-up phase, only HR and RR intervals showed acceptable agreement between the two measuring methods. For all the other measures of HRV, the obtained agreement parameters from the Bland–Altman analysis were higher than the one fixed in Table 1, which shows an inadequate agreement between the two methods. The values of the CC Lin were also less than 0.75, showing at best a moderate agreement. Furthermore, the absolute values of the CC Lin decreased between warm-up and canter phase, showing a decrease in the concordance of the data with the increase of exercise intensity.

## Discussion

Our study shows that when comparing recordings obtained during exercise in horses using an ECG or a HRM, agreement is poor between measures of the HRV analysis, with the exception of HR and RR-interval.

Even with a substantial loss of data due to numerous artifacts leading to poor quality recordings, the analyzed data included a substantial number of comparisons (from 23 horses for the warm-up and 14 for the canter), which is much larger than in previous studies: Parker et al. used 6 horses and Ille et al. used 14 horses (29, 30). The originality of our work also lies in the use of cardiac recordings obtained during exercise, while the previous studies exploited resting recordings.

The results from these studies report comparable results for both devices (29, 30). However, at rest, there are fewer artifacts that the devices have to deal with or that have to be corrected and the RR intervals are larger. This was very well objectified in our study. Recordings are of excellent quality at rest and during the warm-up phase (Figure 2). Their quality deteriorates when horses are moving at higher speed than trot. On the one hand, the movements of the sensors are more important—movements of the skin, muscle contraction, friction with the harness; on the other hand, sensors tend to peel off the skin with sweat. This does not prevent measurement of major phenomena such as HR, but this hinders the signal quality for finer analysis (HRV). When addressing recordings during exercise, a previous study analyzing time-domain and frequency-domain measures of HRV showed a decrease of the agreement with the increase of movement (30). This reduced agreement when speed increase was also seen in our results. Moreover, HR or RR intervals are raw data but are not *sensu stricto* an analysis of HRV that reports the beat-to-beat changes of the successive contractions. For the more sophisticated measures of HRV, LF and HF in normalized units showed moderate agreement in recordings taken during low speed exercise. However, the agreement was not acceptable with the increase of speed during effort accompanied by more artifacts, and all other measures of HRV included in our analysis showed poor agreement for all speeds.

Further, former studies used Pearson’s correlation coefficient or paired *t*-tests for the comparison and this difference in method might also explain differences between their results and our study. In opposition to Pearson’s correlation coefficient, Lin’s coefficient moreover assesses the systematic difference between the data sets, which represents the exactitude of the agreement (40). Pearson’s coefficient assesses then the correlation between two datasets, while Lin’s coefficient really assesses the agreement between two datasets. The Bland–Altman analysis allows to compare data in absence of a definite gold standard and to quantify agreement and to assess agreement even if there are systematic errors in the compared data sets and, therefore, a correlation might still be present even if one method over- or underestimates the true value (39, 42). Furthermore, it allows to have a visual representation of the agreement and an idea of the way the data are—or not—agreeing. For the Bland–Altman analysis, satisfactory agreements were set at 5% of the mean value of the measure of HRV for the first agreement measure (i.e., accepted mean difference) and 20% of the mean value of the measure of HRV for the second measure (i.e., accepted limits of 95% confidence interval). These limits are relatively low and are clinically acceptable for RR and HR that must be very close when taken at the same time on an animal with two different devices (HRM and ECG). There are, however, less data in the literature for the other studied measures of HRV.

Non-concordance between ECG and HRM might also be explained by the difference of sensibility of the devices for detecting the electrical activity of the heart and different algorithms included into the software developed to calculate the HRV (21, 43). An important source of errors might be related to the post processing, especially extensive for data from the HRM (21, 35) and, therefore, reducing considerably the HRV of the HRM-recording. Moreover, the T-wave hyperpolarization is sometimes interpreted by HRM as a QRS signal and leads to erratic beats. Our data from the HRM were corrected in two steps. First, the software provided by the manufacturer was used and after extraction of the RR-intervals the most recognizable abnormalities were corrected. This artificially reduction in the variability of successive RR-intervals might have mostly affected the results for SDNN, RMSSD, LF, and HF, measures calculated directly on the variability of RR-intervals. Influencing only one part of the compared data might have led to a decrease in the agreement with the ECG that were less influenced by post processing and, therefore, might reflect more accurately the real variation in RR intervals. Further, the synchronization was done visually for each pair of recordings by identifying common pattern of RR variation. By doing so, the most comparable parts of the recording were chosen and the chance of agreement of the recordings increased. This might also have affected the results by making the agreement better than it really would be, but as the agreement was generally poor for most of the analyzed measures, this limitation is unlikely to affect the overlap conclusions.

Many studies use either an ECG or a HRM for HRV analysis in the horse; however, the choice of the device used is rarely explained (2, 3, 5–8, 44, 45). According to Ille et al. and Parker et al. (29, 30), HRV analysis at rest can be done indifferently with an ECG or a HRM, the choice of the device being not too important. However, our results show that at exercise, the choice of the device affects greatly the results of the HRV analysis. It is, therefore, important to consider the device used for those studies, and separate the results of studies using HRM (5–10, 45–48) from the ones using ECG. In the future, effort could be made to improve the quality of recordings during exercise. It is essential to find means of limiting the movements of the sensors. This may involve the inclusion of sensors in the harness, girth or blanket, or the use of wireless sensors. The development and miniaturization of connected objects should eventually allow for these improvements.

## Conclusion

This study allowed us to evaluate the agreement on HRV analysis between HRM and ECG during exercise. We concluded to a good agreement for HR and RR intervals but a low agreement for all studied measures of HRV; therefore, comparison of results of HRV for HRM and ECG is difficult. Analyses based on ECG recordings seem to be more accurate and have fewer artifacts than those from HRM. Furthermore, the software included in the ECG device has less post processing options. Yet, corrections and data processing included in the software of the devices affects largely the output used in the subsequent HRV analysis. The HRM can still be used to assess mean heart rate, but analyses of the HRV should be interpreted with caution, especially for recordings obtained during exercise, as they do not agree with the values obtained from ECG recordings. When designing a study on cardiac activity, it is then important to choose the equipment in a reasoned way according to the objective of the study. For recordings at exercise, ECG is preferable if HRV analysis is necessary for the study. For recordings at rest or if only the HR or RR is needed, both types of devices can be used. In addition, efforts should be undertaken to increase standardization for recording, to find ways to maintain good electrode contact during recordings and reduce movement artifacts at trot and canter and to improve the software in order to increase the agreement between both devices.

## Ethics Statement

The study was approved by the Animal Use and Care Committee at Alfort Veterinary School and University of Paris-Est (ComEth Anses/ENVA/UPEC; approval number 12/07/11-1). All the horse owners gave their informed consent on a written and signed document prior to any study procedures.

## Author Contributions

AL: contributed to data collection, data analysis, data interpretation, and wrote the paper. DT: contributed to the experimental design, data interpretation, and wrote the paper. MY: contributed to data collection and data analysis. EB: contributed to the experimental design and data collection. CR: managed the project, experimental design, data collection and data interpretation, and wrote the paper.

## Conflict of Interest Statement

The authors declare that the research was conducted in the absence of any commercial or financial relationships that could be construed as a potential conflict of interest. The reviewer MZ and handling editor declared their shared affiliation.
